# C-reactive protein levels in the perioperative period as a predictive marker of endoscopic recurrence after ileo-colonic resection for Crohn’s disease

**DOI:** 10.1038/cddiscovery.2016.32

**Published:** 2016-05-23

**Authors:** E Iaculli, M Agostini, L Biancone, C Fiorani, A Di Vizia, F Montagnese, S Sibio, A Manzelli, M Tesauro, A Rufini, GS Sica

**Affiliations:** 1 Department of Experimental Medicine and Surgery, University of Rome ‘Tor Vergata’, Rome, Italy; 2 Department of Translational Medicine, University of Rome ‘Tor Vergata’, Rome, Italy; 3 Department of Cancer Studies - CRUK, University of Leicester, Leicester, UK

## Abstract

The aim of this study was to determine the perioperative behavior of C-reactive protein (CRP) in Crohn’s disease (CD) patients undergoing elective ileo-cecal (IC) resection and to identify association between perioperative CRP levels and endoscopic recurrence at 1 year. Study hypothesis was that perioperative CRP changes are disease specific and could detect subset of patients with more aggressive pathopysiology. Seventy-five patients undergoing IC resection for CD were prospectively enrolled. Serial CRP levels were assessed: preoperative, postoperative day 1 (POD1) and day 5 (POD5). CD patients’ values were compared against same interval assessments of control groups undergoing right colectomy and appendicectomy. At POD1, the serum concentration increase was significantly higher in CD patients than in controls. Comparing with control groups, CRP levels remained remarkably high and showed a lower reduction in CD at POD5. Difference between groups was statistically significant. Optimal cutoff levels have been identified: serum CRP concentrations of >39.8 mg/l at POD1 and of >23.2 mg/l at POD5 have shown a significant association to endoscopic recurrence when using bivariate correlation. In this preliminary series, binary logistic regression could not demonstrate statistical relationship between endoscopic recurrence and any of the variables evaluated as prognostic factor. This is the only study so far that investigates and confirms a disease-specific upregulation of CRP response in the perioperative period for CD patients undergoing surgery. The postoperative CRP levels and kinetics seem to be related to the grade of mucosal inflammation and recurrence rate according to our 12 months endoscopic evaluation.

## Introduction

Inflammatory bowel disease (IBD) consists of two major disorders, Crohn's disease (CD) and ulcerative colitis.^1,2^ Although the etiology of IBD is still largely unknown, evidence in many studies indicates that individual’s genetic susceptibility,^3,4^ external environment, intestinal microbial flora and immune responses are all involved in the pathogenesis of IBD. Genetic researches have shown that autophagy, an essential biological process,^5,6^ in immune responses has an indispensable role in IBD. Indeed, two autophagy-related genes named ATG16L1 and IRGM have been reported to be mutated in IBD.^7^ In addition, more recently, small non-coding RNAs (microRNAs),^8^ which have an important role in several biological^9–12^ and pathological processes,^13,14^ are novel players in IBD.^15,16^

In particular, the clinical course of CD is characterized by recurrent episodes of flares followed by complications leading to surgery.^17^ Postoperative recurrence of the disease is unpredictable. However, up to 70–90% of patients show endoscopic recurrence at 1 year.^18,19^

C-reactive protein (CRP) is an acute-phase protein produced by hepatocytes over a context of stress response such as inflammation and infection.^20^ The clinical use of CRP in the management of IBD has been investigated in several studies, and its role in CD pathogenesis has been already postulated.^21^ At hospital discharge after intestinal resection for CD, higher CRP levels are observed in a proportion of patients with no postoperative complications. Such elevated values might reflect a pathologic persistent response and ‘abnormally abnormal’ upregulation of immune system in the context of a surgically-induced remission. This study aims to identify association between perioperative CRP levels in patients undergoing elective ileo-cecal (IC) resection for CD and endoscopic recurrence in a 12-month follow-up.

A second end point is to investigate and compare the kinetics of CRP after IC resection in CD patients against two control groups of ‘non-IBD’ surgical patients, such as acute appendicitis and right colon cancer.

## Results

### Patient cohort

During the study period, 108 patients undergoing ileo-colonic resection for complicated CD were identified. Among these, 79 met the inclusion criteria (group A, CD Group) and were prospectively enrolled in the protocol. Four patients (5%) were excluded after surgery because of early postoperative complications. Therefore, the analysis included 75 consecutive CD patients. All patients underwent protocol follow-up for recurrence evaluation. Sixty-three patients (84%) had a colonoscopy at 12 months. Twelve patients (16%) with no clinical signs of recurrence did not have formal endoscopic evaluation at 1 year because lost at follow-up (7%), lack of compliance to endoscopic protocol or pregnancy.

Demographics and clinical characteristics of the patients considered in the analysis are summarized in Table 1.

### Endoscopic recurrence

**Figure 1:**
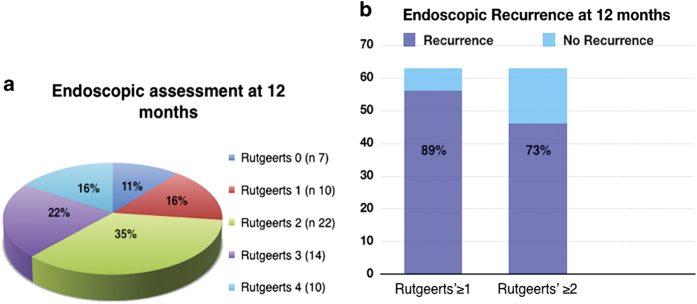
Rutgeerts' score (**a**) and endoscopic recurrence (**b**) at 12-month follow-up. Conventional colonoscopy and ileoscopy are used to assess asymptomatic endoscopic recurrence in CD patients. Rutgeerts' score is used to assess severity of the mucosal inflammation and reactivation of the disease. (**a**) Endoscopic findings at 12-month follow-up (*n=*63) are shown. Lesions graded as Rutgeerts’ ≥1 are traditionally considered compatible with CD recurrence. As operator-dependent, overzealous evaluation can lead to high endoscopic recurrence rate in asymptomatic patients. Recently, a Rutgeerts’ score of ≥2 has been proposed as a new cutoff for this reason. (**b**) The percentage of endoscopic signs of recurrence when considering the two different cutoffs at the same 12-month endoscopic assessment (*n=*63) is shown.

At 12 months, endoscopic recurrence was observed in 56 out of 63 patients (89%) when considering a Rutgeerts’ score of ≥1 as a cutoff value. The mean endoscopic score of recurrence was 2.1. When a Rutgeerts’ score of ≥2 was used as a cutoff value, 46 patients (73%) showed endoscopic signs of recurrence (Figures 1a and b).

### Preoperative CRP serum levels

Before surgery, CRP levels within the normal range (≤5.0 mg/l) were observed in only 19 out of the 75 (25%) CD patients (group A), in 8/50 (16%) patients undergoing appendectomy (group B) and in 22/50 (44%) patients undergoing right colectomy for cancer (group C).

The mean (±S.D.) serum CRP levels before surgery were 27.8 mg/l (±33) in group A, 70.5 mg/l (±65) in group B and 14.2 mg/l (±14) in group C.

**Figure 2:**
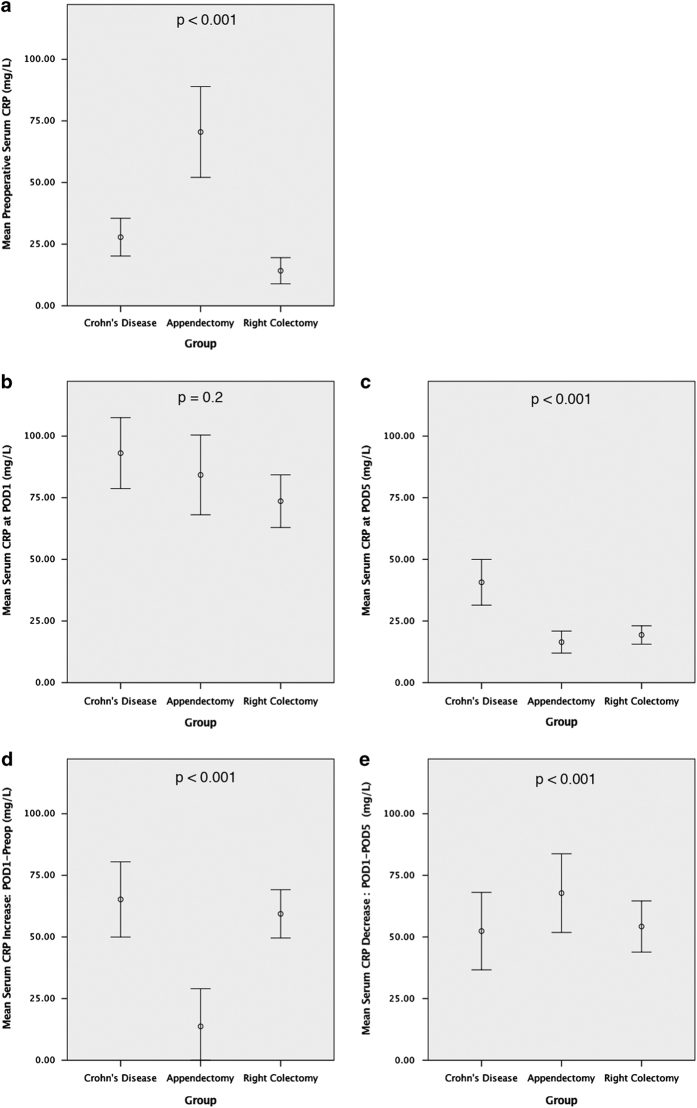
Comparison of perioperative serum CRP levels and kinetics among three surgical groups. CRP is an acute-phase protein characterized by rapid onset and short half-time, and its level and kinetics can be monitored as a marker of inflammatory status in CD patients. CRP levels are measured at three different timepoints (preoperative, POD1, POD5) to evaluate the inflammatory system competence after surgery and to evaluate disease-specific abnormal responses when compared with two control groups of surgical patients. Group B (patients undergoing appendectomy; *n*=50) is considered as a clinical model for CRP production after infective stimulus. Group C (patients undergoing right colectomy; *n*=50) is considered as a clinical model for CRP response to surgical trauma. Comparison between three groups allows to identify differences in CRP levels after surgery at each timepoint (**a**–**c**) and to assess different kinetics in CRP response (**d** and **e**). Bars show mean±S.E.M.; one-way ANOVA test is used for comparison of groups.

As expected, patients undergoing appendectomy showed a mean of preoperative CRP levels significantly higher than patients undergoing surgery for CD or for colon cancer (*P*<0.001) (Figure 2a), with notably higher inter-individual variation as shown by wide 95% confidence interval in the graph.

### CRP serum levels at postoperative day 1

The mean (±S.D.) serum CRP levels at this determination were 93 mg/l (±62) in group A, 84.2 mg/l (±57) in group B and 73.6 mg/l (±29) in group C.

Predictably, all the patients of the cohorts showed high serum CRP levels 24 h after surgery with no difference among groups in terms of absolute values (*P*=0.2) (Figure 2b).

**Figure 3:**
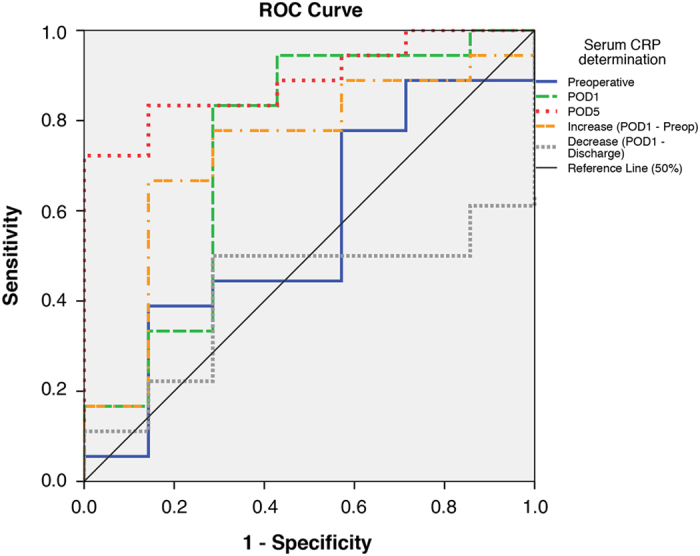
ROC analysis curve for each perioperative serum CRP determination as a predictor of endoscopic recurrence in the test group (*n*=25). The AUC in the ROC analysis is a measure of the diagnostic performance of a test: an AUC value of ≥50 suggests the ability of a test to significantly differentiate between positive and negative outcomes when classifying by determined variable (endoscopic recurrence as with Rutgeerts’ score ≥2). A diagnostic test with an AUC of >0.75 is deemed to have high diagnostic accuracy. To evaluate the overall ability of perioperative CRP as a prognostic marker for CD recurrence at 12 months and to determine the diagnostic cut preliminary ROC analysis has been performed on a test group of the first 25 consecutive patients of the study population. ROC curves for each different determination timepoints using the calculated CRP cutoffs (specified in Table 2) as possible endoscopic predictors (Rutgeerts’ score ≥2) at 12 months in a test group (first 25 consecutive patients of the study population) are shown.

However, when considering the kinetics of CRP, the increase between preoperative and postoperative values was significantly higher in CD than in the other control groups accounting for a stronger CRP response (*P*<0.001) (65.2 mg/l in group A; 13.2 mg/l in group B; 59.3 mg/l in group C) (Figure 3b).

### CRP serum levels at postoperative day 5

At 5 days after surgery, normal CRP levels (±5.0 mg/l) were observed in 4 out of the 75 (5%) CD patients, in 20/50 (40%) patients undergoing appendectomy (group B) and in 6/50 (12%) patients undergoing right colectomy for cancer (group C).

As shown in Figure 2c, the mean (±S.D.) serum CRP levels at postoperative day 5 (POD5) without complication were 40.7 mg/l (±40) in group A, 16.4 mg/l (±15) in group B and 19.3 mg/l (±10) in group C (*P*<0.001).

As a matter of fact, both control groups showed a marked decrease in CRP serum levels, getting close to baseline. On the contrary, CRP levels remained persistently and remarkably high in group A accounting for the lowest fall among groups (52.3 mg/l in group A; 67.8 mg/l in group B; 54.2 mg/l in group C) (Figure 2e), and reducing to normal levels in five patients (10%). The difference between groups was statistically significant (*P*<0.001).

### Serum CRP levels in the perioperative period and CD recurrence

The receiver operating characteristic (ROC) analysis performed in the preliminary test group (*n*=25) of the cohort indicated a significant association (area under the curve (AUC)>0.5) between CRP serum levels at any measurement time and endoscopic recurrence (as with Rutgeerts’ score ≥2) (Figure 3), with optimal cutoff levels for predicting endoscopic recurrence identified as shown in Table 2 with relative sensitivity and specificity.

**Figure 4:**
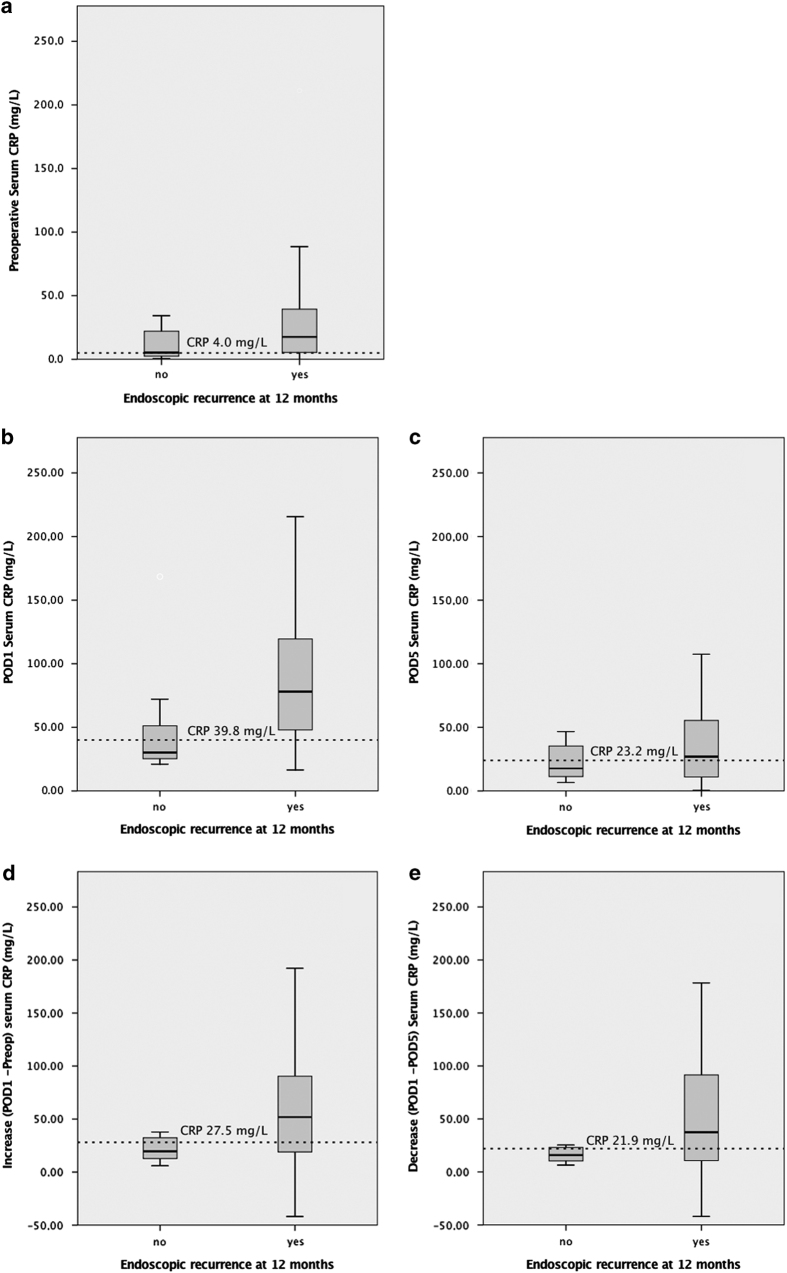
Comparison of serum CRP perioperative levels (mean±S.E.M) in patients with and without endoscopic recurrence at 12 months. The study population has been divided into two subgroups according to the presence of endoscopic recurrence (Rutgeerts' score ≥2) at 12 months. Boxplots show differences between the two subgroups in serum CRP concentration for each determination timepoint (**a**–**c**) and its kinetics (**d** and **e**). The dotted line shows the calculated CRP cutoff used as a prognostic factor for endoscopic recurrence. Calculated CRP cutoffs seem to be able to discriminate between the two groups: patients developing recurrence show higher CRP levels and constantly above the given diagnostic threshold in each determination except for the preoperative one.

These threshold values have therefore been applied for validation in the whole study cohort using bivariate correlation. All but preoperative values identified have been proven statistically significant for the study population of CD patients as demonstrated in Table 3. Figures 4a–e show the different distribution of CRP levels at the given perioperative measurement times in patients who subsequently developed endoscopic recurrence at 1 year and those who did not.

Binary logistic regression was subsequently used to determine the association between clinico-pathological characteristics already known as clinical predictor of disease severity, serum amylase concentration at any perioperative determination (as a dichotomized variable according to cutoff levels) and Rutgeerts’ endoscopic recurrence (score ≥2) at 12 months are shown in Table 4.

## Discussion

Biological biomarkers have a key role in biomedical research as well as in clinical practice.^22,23^ They are now particularly used as surrogate end points in clinical trials,^24,25^ pharmacological treatment^26,27^ of major pathological conditions including cancer^28–32^ and heart disease.^33–35^ Among these, CRP is an acute-phase protein produced by hepatocytes that is upregulated by pro-inflammatory cytokines (for example, interleukin (IL)-1, IL-6 and tumor necrosis factor (TNF)-a) in the context of mainly Th-1 response. Its biological role *in vivo* is still under investigation, but it is assumed to be involved in innate immune response and complement cascade activation.^36–38^ Comparing with other bio-humoral markers, CRP has peculiar characteristics potentially useful in clinical practice: rapid production (6–12 h) in response to acute inflammatory processes; short half-life (48–72 h); serum level not influenced by medical therapies.^39^ As a result, its robust relationship with clinical events is mostly used in differential diagnosis, follow-up, monitoring medical treatment and prediction of clinical course in several conditions.^38^ Accordingly, the use of serum CRP levels as a prognostic factor has proved to be effective in order to improve clinical management (for example, acute pancreatitis, myocardial infarction, myeloma and IBD).^37,40^

The role of CRP in CD has been largely investigated: to detect and differentiate IBD from irritable bowel syndrome,^41^ to monitor disease activity, to evaluate treatment response^42,43^ and to predict disease course.^17,37^ There is clear correlation between CD and increased CRP serum concentration and although its use in predicting clinical relapse and postoperative recurrence has been suggested,^44^ the value of CRP levels after surgery as a prognostic factor has not been investigated as yet. This is the first study to document the association between serum CRP concentration at the time of IC resection and endoscopic recurrence in CD patients.

Specifically at our best knowledge no existing studies have addressed the specific behavior of CRP in CD patients after surgery and its clinical relevance. A strong CRP response is widely documented in CD^37,38,45^ and commonly used in clinical practice for diagnosis and management. In CD patients undergoing surgery, persistently high levels of CRP may be observed at hospital discharge in a subgroup even in the absence of overt surgical complications. This finding might account either for an uncontrolled activation of the immune system as previously reported^46,47^ or be related to an increased production of acute-phase mediators due to surgery. On the other hand, CRP levels after surgery may widely differ from patient to patient regardless of the surgical trauma: in our study, high CRP levels are not observed in all patients at hospital discharge (95% in our study). Of note, wide interval in serum concentrations is recorded (POD5: min 0.7 mg/dl–max 227.7 mg/dl).

To determine the peculiar effect of CD on CRP changes after surgery and also to weigh-up the influence of residual systemic inflammation due to surgery or infection, we compared the study population with two control groups of surgical patient randomly chosen from our databases: group B, right colectomy for cancer (*n*=50) and group C, appendectomy for acute appendicitis (*n*=50). With such an investigative strategy, perioperative changes in CD were contrasted against the body systemic response after plain surgical insult (group B) and against the possible residual inflammation due to infection (group C). Considering the wide inter-individual variation and the multiple factors influencing CRP production, such a study design intends to reproduce a clinical model to ponder ‘CD effect’, ‘surgery effect’ and ‘infection effect’ upon the perioperative CRP levels.

Our results corroborate the preliminary observation of higher CRP serum levels in CD patients undergoing elective uncomplicated IC resection comparing with other surgical patients. Particularly, the kinetics of this inflammatory marker showed a significantly higher (*P*<0.001) delta between CRP levels before and after the surgical insult. Likewise, the CRP assessment at 5 days after procedure confirms a persistent upregulation of the acute-phase protein that is significantly different from control groups (*P*<0.001). This proven sustained stimulation in CD patients might therefore be implicated in disease pathogenesis and correlate with its clinical severity and with risk of recurrence as postulated in the primary end point of this study.

In this respect, CRP is a well-known non-invasive surrogate marker of mucosal inflammation and it has been found helpful to predict early clinical relapse during disease remission.^46,48^ This observation has been confirmed by several independent studies.^37,49^ With this assumption and following evidence of a CD-specific response to surgical procedure as indicated by our results, the CRP profile in each patient could be used as a preclinical marker for recurrence as soon as the immediate postoperative days. Abnormal perioperative CRP profile can proportionally reflect an upregulation in the immune system and host response in CD accounting for a more aggressive disease with higher risk of recurrence. Predicting the incidence and the severity of recurrence in CD would lead to identification of high-risk patients to be considered for intensive maintenance therapy and strict follow-up.

Evaluation of our study cohort shows that the rate of endoscopic recurrence (Rutgeerts’ score ≥1) in our series at 1 year was high at 89% (median score 2). This possibly reflects rigorous examination and perhaps overestimation of earlier lesions during endoscopy (16% Rutgeerts' score 1). When a higher cutoff value (Rutgeerts’ score ≥2) was used to assess endoscopic recurrence, the observed rate (73%) was similar to that reported by the current literature.

In our study cohort, serum CRP concentrations of >39.8 mg/l at postoperative day 1 (POD1) and of >23.2 at POD5 show a strong association with Rutgeerts’ severity score ≥1 (*P=*0.08 and *P=*0.03), likewise a brisk increase in CRP level >27.5 mg/l after surgery (*P=*0.03). On the other hand, in a regression analysis we have not been able to demonstrate the statistical utility of perioperative serum CRP levels to predict relapse. This might be related to the limited size of the study group for multivariate association analysis as suggested by the lack of significance for other clinico-pathological variables already acknowledged to be independent risk factor for recurrence.^50,51^

Potential limitations of the study should be taken into account. The main drawback of this single-center prospective longitudinal trial is the relatively limited cohort of patients evaluated for recurrence (*n*=63) accounting for the absence of group stratification and lack of statistical power also for well-known CD-recurrence risk factors. Finally, several individual genetic factors might lead to wide inter-individual variation in CRP response.^37,39^

## Conclusions

This is the only study so far that investigates CRP postoperative modifications in CD patients. Our preliminary data confirm a disease-specific activation and upregulation of CRP response in CD patients after surgery. We suggest that the degree of immunologic changes and related severity of disease might be explored immediately soon after surgery by determining perioperative CRP modification.

The postoperative CRP levels and kinetics as calculated in our cohort are statistically related to the grade of mucosal inflammation and recurrence rate according to our 12-month endoscopic evaluation.

The individual variability of CRP activation in CD patients and the complex interactions of other prognostic factors for CD recurrence should be acknowledged. Therefore, larger studies with multivariate analyses are needed to validate serum CRP thresholds that might inform risk stratification. This would potentially lead toward implementation of a clinical risk scoring to identify CD patients with more aggressive disease for specific recurrence surveillance as early as the immediate postoperative period.

## Patients and Methods

### Study protocol

In a prospective longitudinal study, all CD patients under regular follow-up in a single referral center undergoing elective ileo-colonic resection as previously described^52^ were enrolled (group A, *n*=75). Fifty patients having appendectomy (group B) and fifty patients having right colectomy for colon cancer (group C) during the same study period were randomly chosen as controls from a surgical database. All the procedures were performed laparoscopically or as open surgery according to surgeon’s preference.

For each patient of the three groups in the serum CRP levels were measured at the same referral laboratory at three fixed perioperative times: before surgery (within 7 days), at POD1 and at POD5 from procedure (before or after hospital discharge). All samples were measured by enzymatic immunoassay method with a normal range of 0–5 mg/l.

After surgery, all CD patients were treated with mesalazine (2.4 g/day) within 1 month from resection and were prospectively followed up for 1 year.^53^

Endoscopic recurrence was assessed by conventional colonoscopy at 12 months, according to the Rutgeerts’ score (grade 0–4).^51,54–56^ Recurrence at endoscopy was defined as Rutgeerts’ score ≥1.

Patients consent to participate in the study and in the relative follow-up protocol was obtained at the time of consent for surgical procedure.

The study design was approved by the ‘Policlinico Tor Vergata’ Trust Ethics Committee.

### Patients

In CD patients (group A), inclusion criteria were as follows: (1) diagnosis of CD according to conventional criteria;^57^ (2) regular follow-up at the tertiary referral IBD center of the Policlinico ‘Tor Vergata’ of Rome, Italy; (3) age ≥15 and 70≤ years; (4) disease involving the distal ileum or both the distal ileum and right colon; (5) indication for elective IC resection; (6) resection performed in the same gastrointestinal Surgical Unit; (7) agreement to the study protocol.

Exclusion criteria included were as follows: (1) use of immunomodulators within 30 days from surgery; (2) emergency surgery; (3) active perianal disease; (4) additional major surgical procedures; (5) postoperative complications including wound infection; (6) concomitant chronic inflammatory diseases, immunologic disease, chronic infections, serum creatinine levels >1.5 mg/dl or severe comorbidities.

### Statistical analysis

All the statistical analyses were performed using SPSS for Windows, Version 16.0. (SPSS Inc, Chicago, IL, USA). Descriptive outcomes were expressed as mean and standard deviation or frequency and percentages. Quantitative data showing normal distribution were analyzed by the Student's *t*-test. Serum CRP levels were compared between groups by the one-way ANOVA procedure when equal variance was assumed, while the Kruskal–Wallis’ non-parametric test for independent samples was used when data were not normally distributed.

An ROC analysis was performed in an initial test cohort of consecutive patients (*n*=25) to assess the diagnostic power between serum CRP levels for each perioperative measurement and endoscopic recurrence (Rutgeerts’ score ≥2). Relative sensitivity and specificity are evaluated to identify the best threshold values of serum CRP as possible predictors.

Bivariate correlation between each perioperative CRP measurement (as a dichotomized variable) and endoscopic recurrence (as Rutgeerts’ score ≥2) was used in the whole study group (*n*=75) to validate threshold values as a prognostic factor. Association analysis between the severity of endoscopic recurrence and clinical predictor variables including perioperative CRP serum levels was measured using binary logistic regression.

All tests were two-tailed analyzed. Regardless of the statistical analysis used, *a P*<0.05 value was considered as statistically significant.

## Figures and Tables

**Table 1:** Patients demographics (n=75)

*Demographics*	n*=75*
*Gender*	
Male	39 (52%)
Female	36 (48%)
	
Age (mean±S.D.; years)	42 (±11)
	
*Smoking habit*	
Non-smoker	30 (40%)
Smoker	27 (36%)
Ex smoker	18 (24%)
	
CD duration (mean ±S.D.; years)	10(±7)
Age at diagnosis (mean ±S.D.; years)	31 (±11)
	
*CD behavior*	
Fibrostricturing	55 (73%)
Fistulizing	16 (21%)
Stricturing-fistulizing	4 (6%)
	
*Medical treatment before surgery*	
No therapy	14 (19%)
Mesalazine	19 (25%)
Corticosteroids	23 (30%)
Budesonide	14 (19%)
Mesalazine and Budesonide	5 (7%)
	
Previous CD surgery	27 (36%)
	
*Surgical technique*	
Laparoscopy	41 (55%)
Open	34 (45%)

**Table 3:** Bivariate correlation between calculated serum CRP threshold values and Rutgeerts’ recurrence at 12 months (*n*=63)

*Calculated serum CRP threshold values*	*Correlation with endoscopic recurrence (*n*=63*)
CRP preop 4.0 mg/l (Figure 4a)	*P=*0.15
CRP postop (POD1) 39.8 mg/l (Figure 4b)	*P=*0.008
CRP discharge (POD5) 23.2 mg/l (Figure 4c)	*P*=0.03
CRP increase (POD1−Preop) 27.5 mg/l (Figure 4d)	*P=*0.03
CRP decrease (POD1−POD5) 21.9 mg/l (Figure 4e)	*P*=0.07

Spearman’s rank correlation coefficient is used.

**Table 4:** Association between perioperative CRP levels, clinico-pathological risk factors and Rutgeerts’ score at 12 months (*n*=63)

*Recurrence risk factors*	*Association with Rutgeerts’ score at 12 months (*n*=63)*
CRP preop	*P=*0.91
CRP postop (POD1)	*P=*0.71
CRP discharge (POD5)	*P=*0.36
CRP increase (Preop−POD1)	*P=*0.08
CRP decrease (POD1−POD5)	*P=*0.13
Smoking habit	*P=*0.93
Gender	*P=*0.92
CD behavior	*P=*0.86
CD duration	*P=*0.90
Age at diagnosis	*P=*0.73
Treatment before surgery	*P=*0.70

Binary logistic regression analysis is used.

**Table 2:** Serum CRP level: prognostic cutoffs calculation

*Serum CRP level (test group,* n*=25*)	*AUC (%)*	*Calculated threshold value (mg/l)*	*Sensitivity (%)*	*Specificity (%)*
CRP preop	55	4.0	67	58
CRP postop (POD1)	73	39.8	80	72
CRP discharge (POD5)	88	23.2	86	72
CRP increase (Postop−Preop)	73	27.5	74	72
CRP decrease (Postop−Discharge)	42	21.9	60	72

ROC analysis is performed in a test group (*n*=25) of the study population to assess diagnostic accuracy of each perioperative measurement. Threshold values are calculated with relative sensibility and specificity as a prognostic marker for endoscopic recurrence in the test group.

## Abbreviations

AUC: area under the curve
CD: Crohn’s disease
CRP: C-reactive protein
IC: ileo-cecal
IBD: inflammatory bowel disease
IL: interleukin
POD: postoperative day
ROC: receiver operating characteristic
TNF: tumor necrosis factor.
